# Unlocking the Potential of Rucaparib: A Case Series on Its Impact in Metastatic Breast Cancer With Mutations

**DOI:** 10.7759/cureus.60963

**Published:** 2024-05-23

**Authors:** Chandrakanth MV, Vivek Agarwala, Neha Choudhary, Amit Sharma, Minakshi Roy, Kaustav Mandal, Moinak Basu, Nibedita Sen, Pritam K Sarkar, Subhabrata Kumar

**Affiliations:** 1 Medical Oncology, Narayana Superspeciality Hospital, Howrah, IND; 2 Medical Oncology, Rabindranath Tagore Hospital, Kolkata, IND; 3 Medical Oncology, Narayana Superspeciality Hoapital, Howrah, IND; 4 Radiotherapy, Narayana Superspeciality Hospital, Howrah, IND

**Keywords:** target therapy oncology, parp-inhibitors, rucaparib, brca 1/2, metastatic triple-negative breast cancer

## Abstract

Triple-negative breast cancer poses distinct challenges because it lacks hormone receptors and does not have human epidermal growth factor receptor 2 (HER2) amplification. Mutations in BRCA1/2 genes are associated with homologous recombination deficiency tumors, rendering them susceptible to poly (ADP-ribose) polymerase (PARP) inhibitors. Notably, germline BRCA1/2 mutations are linked to distinct clinical features, including an increased risk of triple-negative breast cancer (TNBC) and a younger age of onset. PARP inhibitors such as olaparib and talazoparib have demonstrated efficacy in patients with BRCA mutations, leading to FDA approvals for ovarian and breast cancers. However, there remains limited data on PARP inhibitor response rates in patients with somatic BRCA mutations. This case series demonstrates the use of rucaparib in metastatic breast cancer patients harboring both germline and somatic BRCA1/2 mutations, discussing the advancing landscape of targeted therapies in breast cancer management. In the first case, despite undergoing anthracycline-based chemotherapy followed by hormonal therapy, disease progression ensued. However, transitioning to rucaparib yielded a remarkable complete response lasting over two years, highlighting its efficacy in this clinical setting. Similarly, in the second case, rucaparib demonstrated effectiveness as a maintenance therapy subsequent to achieving a near-complete response to taxane and platinum-based treatment. These findings emphasize the promising role of rucaparib in managing metastatic breast cancer in patients with BRCA1/2 mutations, further contributing to the expanding armamentarium of targeted therapies in breast cancer care.

## Introduction

Breast cancer stands as the most prevalent cancer type among Indian women, with statistics revealing a new diagnosis occurring every four minutes. Over half of affected Indian women are diagnosed at stage 3 or 4 of the disease. Survival rates post-cancer for Indian women are reported at 60%, contrasting with the 80% rate seen in the US [1]. Constituting 15%-20% of all breast cancers (BCs), triple-negative breast cancer (TNBC) is characterized by the absence of estrogen and progesterone receptors and the non-amplification of the ERBB2 gene, which encodes human epidermal growth factor receptor 2 (HER2) [2]. Recent advancements in sequencing technology have provided significant insights into various BC types, including TNBC, at genomic and epigenomic levels. Common occurrences in TNBC include mutations in TP53, loss-of-function in breast cancer susceptibility genes (BRCA) 1, and amplification with heightened expression of myelocytomatosis (MYC) proto-oncogene [3]. Breast cancer may exhibit genetic alterations in the homologous recombination repair (HRR) pathway, which is crucial for double-stranded DNA break repair. Such alterations, including mutations in BRCA1/2, lead to genomic instability and the development of homologous recombination deficiency (HRD) tumors. Mutations in BRCA1/2 genes are pivotal for DNA double-strand break repair via homologous recombination, predisposing individuals to breast and other cancers [4]. Poly (ADP-ribose) polymerase (PARP) plays a significant role in base excision repair, crucial for DNA single-strand break repair. Exploiting dependency on base excision repair for therapeutic purposes, PARP inhibition in BRCA1/2-mutated cancer cells induces unresolved DNA damage, leading to cell death [5].

Patients with germline BRCA1/2 mutations often exhibit distinct clinical features, including an increased risk of developing TNBC and presenting with the disease at a younger age. Additionally, somatic BRCA1/2 mutations have been associated with resistance to chemotherapy and hormonal therapy, suggesting potential benefits from alternative treatment approaches such as PARP inhibitors [6]. The efficacy of PARP inhibitors has been instrumental in improving disease-free survival time. Olaparib (NCT02000622) and Veliparib (NCT02163694) are among the most commonly researched United States (US) Food and Drug Administration (FDA)-approved drugs in this class [7,8]. Olaparib, an oral PARP inhibitor, is approved for treating patients with recurrent ovarian cancer and a BRCA mutation, showing clinically meaningful benefits in such cases. It has also demonstrated promising activity in patients with metastatic breast cancer and a germline BRCA mutation. The OlympiAD trial compared olaparib's efficacy and safety with standard single-agent chemotherapy among HER2-negative metastatic breast cancer patients with a germline BRCA mutation. The median overall survival (OS) was 19.3 months with olaparib compared to 17.1 months with treatment of physician's choice [9].

FDA approval was granted for talazoparib's use in adult patients with germline breast cancer mutation (gBRCAm) HER2-negative locally advanced or metastatic breast cancer, based on findings from the phase III EMBRACA trial (NCT01945775). This trial revealed a statistically significant improvement in progression-free survival with talazoparib compared to the physician's choice of non-platinum-based single-agent chemotherapy [10].

PARP inhibitor monotherapy shows promising efficacy in breast cancer, with early-stage trials demonstrating progression-free survival (PFS) of 7, 28.7, and 22.1 months for olaparib, rucaparib, and niraparib, respectively. Recent phase III studies with olaparib and talazoparib in advanced breast cancer have led to the first regulatory approval of a PARP inhibitor for this indication [11,12]. It was found in a study by Fallah et al. on olaparib in combination with abiraterone that the FDA concluded that the modest PFS combined with the clinically significant toxicities did not reveal a favorable risk/benefit ratio [13]. Rucaparib has been approved by the US FDA for the treatment of patients with deleterious BRCA mutation-associated advanced ovarian cancer who have been treated with two or more therapies and recently by the FDA for BRCA mutation-associated metastatic castration-resistant prostate cancer [14].

Research on PARP inhibitors has primarily focused on patients with germline BRCA mutations, resulting in a lack of data comparing response rates to PARP inhibitors in patients with somatic versus germline BRCA mutations. This case series discusses the utilization of rucaparib in metastatic breast cancer (MBC) patients with both germline and somatic HER2- BRCA1/2 mutations.

## Case presentation

First case

A 37-year-old female, diagnosed with metastatic breast cancer three years ago, presented with an 8-cm mass in her left breast and matted axillary lymph nodes. The core biopsy from the left breast revealed stage III invasive ductal carcinoma (IDC). Immunohistochemistry showed weak estrogen receptor (ER)/partial response (PR) positivity and HER2 negative, with a high Ki67 (60%). A PET-CT scan reported multiple bone metastases. The patient also had a gBRCA1m.

**Figure 1:**
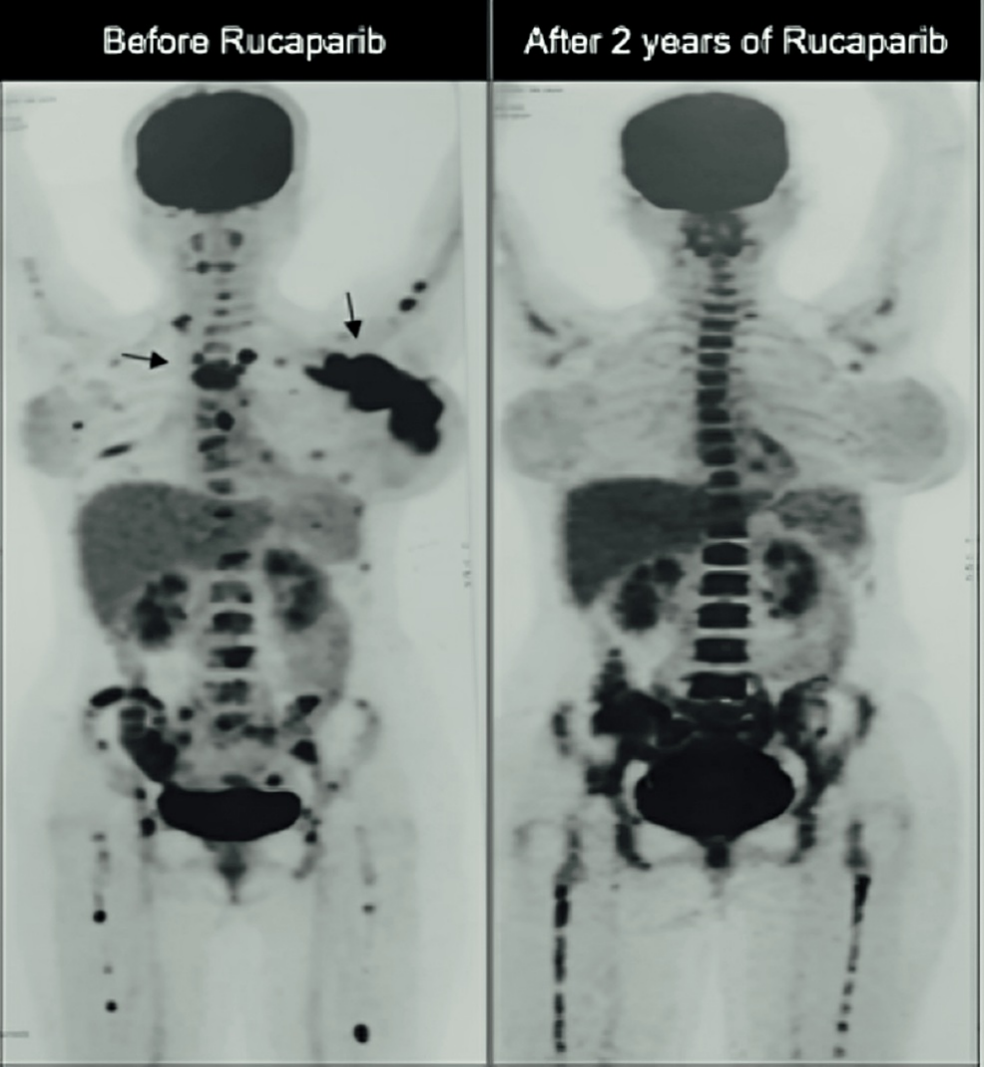
Case 1: Rucaparib Response on PET-CT Scan in BRCA-Mutated MBC Patient BRCA, breast cancer gene; MBC, metastatic breast cancer; PET-CT, positron emission tomography-computed tomography.

Initially, the patient was treated with anthracycline-based chemotherapy due to the weak ER-positive disease. Epirubicin 100 mg/m² IV, 5-fluorouracil 500 mg/m² IV, and cyclophosphamide 500 mg/m² IV therapy were given in a repeated cycle for 21 days. After six cycles, there was a partial response. Subsequently, tamoxifen and ovarian function suppression were initiated, but within three months, there was clinical progression with an increase in the size of the breast lump and new axillary and cervical lymph nodes. This progression was also confirmed by a PET-CT scan. The patient was then switched to rucaparib (Nuparp®) 600 mg bis in die-twice daily (BD), which was later reduced to 300 mg BD due to asthenia and anemia. Currently, the patient is in complete response and has been on a low dose of 300 mg BD of rucaparib for more than two years (Figure 1).

Second case

**Figure 2:**
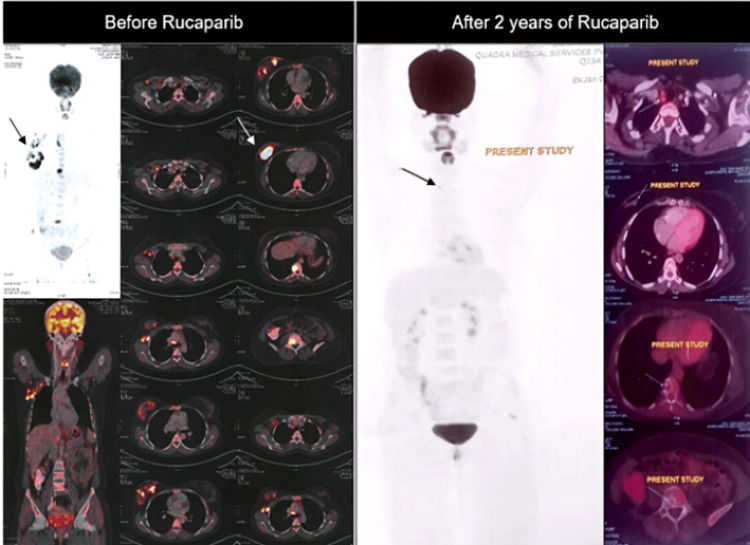
Case 2: sBRCA-Mutated MBC, Two Years Rucaparib Response on PET-CT Scan sBRCA, somatic breast cancer-mutated gene; MBC, metastatic breast cancer; PET-CT, positron emission tomography-computed tomography.

**Figure 3:**
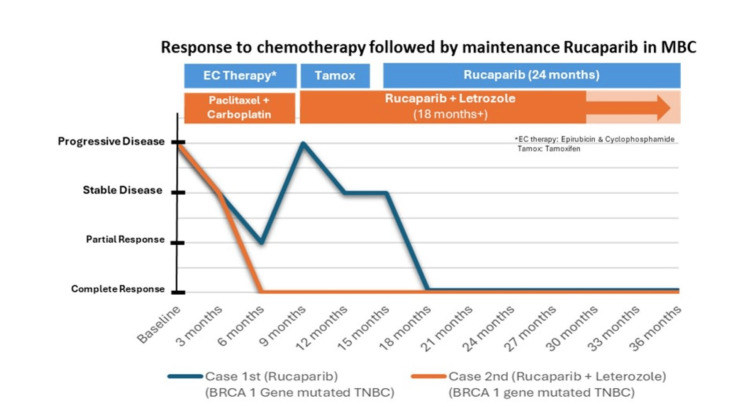
Treatment Response of BRCA1-Mutated TNBC Cases (Case 1 (Blue), Case 2 (Orange)) BRCA1, breast cancer gene 1; TNBC, triple-negative breast cancer; MBC: metastatic breast cancer.

A 35-year-old female was diagnosed with upfront metastatic breast cancer. The IDC was classified as grade III (5+/2+/2+), with no HER2 amplification seen on fluorescence in situ hybridization (FISH). Genetic testing revealed both somatic and germline BRCA2 mutations. Her disease sites included right breast nodules, axillary lymph nodes, and bone metastases. Initial treatment consisted of a combination of taxane and platinum-based therapy, resulting in a near-complete response; the patient was administered with paclitaxel 80 mg/m^2^ and carboplatin on days 1, 8, and 15 every 28 days for six cycles. Subsequently, she was placed on maintenance therapy with rucaparib (Nuparp®) 600 mg PO BD daily along with hormonal therapy Letrozole 2.5 mg PO OD daily and Zoledronic acid once every three months. She has been on this regimen for two years, with monthly monitoring of her hematological parameters. Despite this, she has been doing well and has maintained a complete response throughout the two years of treatment (Figures 2, 3).

## Discussion

The development of poly (ADP-ribose) polymerase inhibitors (PARPi) represents a novel approach to cancer treatment, targeting PARP1, PARP2, and PARP3 proteins crucial in repairing single-strand DNA breaks. As BRCA mutations are further characterized, and more PARPi are developed, therapeutic options for advanced ovarian and certain breast cancers expand. The cases discussed in this series highlight the efficacy of rucaparib in managing metastatic breast cancer (MBC) patients with BRCA1/2 mutations, whether germline or somatic. In clinical practice, combination chemotherapy is typically reserved for MBC with a significant disease burden and weak hormone-positive disease. Tamoxifen, a selective estrogen receptor modulator (SERM), is commonly used as an endocrine therapy in premenopausal women, often combined with ovarian suppression function to treat estrogen receptor (ER)-positive tumors [15]. Case 1 highlights the challenge of managing weak ER-positive disease, where initial anthracycline-based chemotherapy yielded only a partial response. The patient initially presented with an 8-cm lump in her left breast and matted axillary lymph nodes and bone metastases indicative of stage IV breast cancer. Despite subsequent treatment with tamoxifen and ovarian function suppression, there was disease progression. In response to disease progression, a treatment switch to rucaparib was initiated at a dosage of 600 mg twice daily (BID). However, due to adverse effects, including asthenia and anemia, the dosage was later reduced to 300 mg BID. Rucaparib demonstrated effectiveness, achieving a complete response with sustained clinical and radiological improvements. Notably, dose adjustments were necessary to manage adverse effects.

In the latest National Comprehensive Cancer Network (NCCN) guidelines, triple-negative breast cancers (TNBCs) are typically managed with a combination of taxane and anthracycline-based chemotherapy regimens. TNBC often exhibits BRCA gene mutations, rendering them particularly sensitive to DNA-damaging agents like platinum drugs. Various neoadjuvant clinical trials have explored the impact of adding platinum to standard chemotherapy [16]. For instance, a meta-analysis by Pathak et al. in 2022 showed a better partial clinical response (pCR) rate (OR = 2.11; 95% CI = 1.44-3.08; I2 = 67%, p = 0.009) in the carboplatin arm among TNBC patients. Similarly, the GeparSixto trial in 2014 demonstrated a significant improvement in pCR with carboplatin among stage II or III TNBC patients [17,18]. In the presented case, the patient was diagnosed with upfront metastatic breast cancer (MBC) and harbored both somatic and germline BRCA2 mutations. Initial treatment with taxane and platinum-based therapy resulted in a near-complete response, highlighting the efficacy of DNA-damaging agents in this subtype of breast cancer. Following the near-complete response, the patient transitioned to maintenance therapy, incorporating rucaparib, hormonal therapy with letrozole, and zoledronic acid. This maintenance regimen was well-tolerated, allowing the patient to maintain a complete response for two years.

The inclusion of rucaparib as part of maintenance therapy highlights its role not only in achieving an initial response but also in prolonging disease control. Notably, the results of the randomized, phase 3 OlympiAD trial demonstrated that oral olaparib monotherapy significantly prolonged median progression-free survival compared to standard chemotherapy among patients with HER2-negative metastatic breast cancer and a germline BRCA mutation. Additionally, the olaparib group exhibited a response rate approximately twice as high as the standard therapy group, further supporting the efficacy of PARP inhibitors in this patient population [9]. To our knowledge, there are no case reports on the efficacy of rucaparib in BRCA-mutated metastatic breast cancer from the Indian subcontinent.

The results of the RUBY trial provide significant insights into the use of rucaparib in metastatic breast cancer (MBC) patients with BRCA1/2 mutations. Despite not meeting the primary endpoint, which was a clinical benefit rate (CBR) of 13.5%, a subgroup of patients showed notable responses to rucaparib therapy. Particularly noteworthy were the complete and sustained responses observed in two patients lacking somatic BRCA1/2 mutations but exhibiting high levels of genomic loss of heterozygosity (LOH). These responses lasted 12 and 28.5 months, suggesting the potential of LOH as a predictive biomarker for rucaparib efficacy. Furthermore, analysis of whole-genome data revealed a trend associating higher HRDetect scores with treatment response, although this trend lacked statistical significance. Additionally, among a broader cohort of metastatic breast cancer patients screened for LOH, a high LOH score was correlated with a 39% increased likelihood of death (hazard ratio = 1.39, 95% CI = 1.11-1.75, p = 0.005) [19].

These findings underscore the importance of molecular profiling in guiding treatment decisions for MBC patients, particularly those with BRCA1/2 mutations. The positive outcomes observed with rucaparib highlight its potential as a valuable therapeutic option in this subset of patients, either as a monotherapy or in combination with other agents. Moreover, the ability to sustain treatment response and maintain quality of life over an extended period emphasizes the evolving role of rucaparib in personalized medicine for metastatic breast cancer. The case series is constrained by its small sample size of just two patients, which may impact the generalizability of the results. Additionally, while the study includes a follow-up period of two years, more extensive follow-up would be beneficial to fully assess the durability of the treatment response and the long-term safety of rucaparib.

## Conclusions

The presented cases underscore the evolving role of rucaparib in the management of metastatic breast cancer (MBC), particularly in patients with both germline and somatic BRCA mutations. In the first case, the patient exhibited clinical progression despite initial anthracycline-based chemotherapy and subsequent hormonal therapy. However, switching to rucaparib resulted in a remarkable, complete response lasting over two years, highlighting its efficacy in this setting. Similarly, in the second case, rucaparib demonstrated effectiveness as maintenance therapy following a near-complete response to taxane and platinum-based therapy. These promising initial findings encourage further exploration in larger-scale studies to validate and extend the understanding of rucaparib's therapeutic potential in breast cancer treatment.
